# Enhancing Heart Failure Management: A Systematic Review and Meta-Analysis of Continuous Remote Telemedical Management vs. In-Person Visit in Patients with Cardiac Implantable Electronic Devices

**DOI:** 10.3390/jcm14124278

**Published:** 2025-06-16

**Authors:** Boglarka Veres, Boldizsar Kiss, Peter Fehervari, Marie Anne Engh, Peter Hegyi, Endre Zima, Bela Merkely, Annamaria Kosztin

**Affiliations:** 1 Heart and Vascular Center, Semmelweis University, 68 Városmajor utca, 1122 Budapest, Hungary; boglarka.sara.veres@gmail.com (B.V.); b.kiss96@gmail.com (B.K.); zima.endre@gmail.com (E.Z.); merkely.study@gmail.com (B.M.); 2 Center for Translational Medicine, Semmelweis University, 1085 Budapest, Hungary; fehervari.peter.biomat@gmail.com (P.F.); marieaengh@gmail.com (M.A.E.); hegyi2009@gmail.com (P.H.); 3 Department of Biostatistics, University of Veterinary Medicine, 1078 Budapest, Hungary; 4 Institute of Pancreatic Diseases, Semmelweis University, 1083 Budapest, Hungary; 5 Institute for Translational Medicine, University of Pécs Medical School, 7624 Pecs, Hungary; 6 Institute of Anesthesiology and Perioperative Care, Semmelweis University, 1082 Budapest, Hungary

**Keywords:** remote monitoring, cardiac implantable electronic device, heart failure, meta-analysis

## Abstract

**Background/Objectives:** Remote telemedical management (RTM) in heart failure (HF) patients with cardiac implantable electronic devices (CIED) is a reliable approach to follow device-specific and heart failure-related parameters. However, while some positive outcome data is available, results are inconclusive. We aimed to assess the benefits of continuous remote telemonitoring (RTM) compared to the in-person visit (IPV) in reducing all-cause mortality, heart failure hospitalizations (HFH), cardiovascular (CV) deaths, and the occurrence of inappropriate therapy. **Methods:** The study comprised a systematic review and meta-analysis of randomized controlled trials (RCTs) testing RTM (device-related or other non-invasive telemonitoring systems) vs. IPV for the management of HF patients. The main endpoints were all-cause and CV mortality. Risk of bias and level of evidence were assessed. Hazard ratios (HRs), odds ratios (ORs) and 95% confidence intervals (CI) were calculated. CENTRAL, EMBASE and MEDLINE were searched, and only randomized controlled studies were included. **Results:** Sixteen RCTs were identified, comprising a total of 11,232 enrolled patients. Seven studies evaluated all-cause mortality, resulting in an OR 0.83 (95% CI 0.72 to 0.96). When CV mortality was assessed, the RTM group showed a significant benefit compared to the IPV group (OR 0.81, 95% CI 0.67 to 0.97). The risk of bias ranged from “low” to “some concerns” for most outcomes, and the certainty was low to moderate depending on the specific outcomes. **Conclusions:** RTM proved to be superior in reducing all-cause and CV mortality compared to IPV; however, there is a clear need to have standardized alert actions to achieve the mortality benefit.

## 1. Introduction

Heart failure is a complex clinical syndrome leading to frequent hospitalizations and accompanied by a high rate of mortality [1]. Telemedical management (RTM) in this population has proven to be a reliable tool for facilitating the optimization of medical therapy, detecting the early signs of decompensated heart failure (HF) and detecting device-related malfunctions [2,3]. However, RTM remains underused among the 64 million HF-affected patients, whose care could be extended by these devices [1].

Despite the increasing number of randomized controlled trials (RCTs) and observational studies in the field, heterogenous patient populations and unstandardized methods present uncertainty regarding patient outcome and the general use of RTM [4], which is reflected in the current ESC guidelines [5,6]. At the same time, the most recent consensus statement published in 2023 recommends the use of RTM as part of the standard of care with an IA indication [7]. Despite these recommendations, the proportion of CIED patients followed by RTM remains low [8,9].

The IN-TIME trial revealed that in HF with reduced ejection fraction (HFrEF) patients eligible for implantable cardioverter defibrillators (ICD) or cardiac resynchronization therapy (CRT) implantation, RTM decreased the risk of mortality as compared to in-person visits [10].

Other observational studies also confirmed that RTMs led to earlier interventions and related better outcomes, reduced healthcare costs and lowered the burden of frequent clinic and emergency visits—observed as especially beneficial during the COVID-19 pandemic [4,8,11]. However, while telemonitoring can lead to reductions in hospital admissions, all-cause mortality and emergency department visits in some studies, other studies showed no such benefits [4]. Altogether, contradicting results were published, indicating that careful analysis of trial results is needed to determine the overall benefit for HF patients and a standardized workflow.

We aimed to review and analyze the current literature on complex telemonitoring systems (modality in which multiple TM is combined with structured telephone support (STS) and/or access to a 24 h call center or a mix of other sub-modalities) and device-related non-invasive remote monitoring in HF patients to assess whether the use of telemedical care leads to a reduction in all-cause mortality, cardiovascular-related mortality, cardiovascular or heart failure hospitalization rates and number of ICD shocks or inappropriate ICD shocks, as compared to in-person visit.

## 2. Materials and Methods

A systematic review and meta-analysis was reported in accordance with the Preferred Reporting Items for Systematic Reviews and Meta-Analyses (PRISMA) statement, and conducted in accordance with the recommendations of the Cochrane Collaboration. The review protocol was registered on the PROSPERO International Prospective Register of Systematic Reviews (CRD42022299820). Deviations from protocol were made to include a broader range of patients, with the aim of enhancing generalizability. Accordingly, studies following up patients with complex telemonitoring systems were included; however, only one such additional study met the criteria.

### 2.1. Search Strategy

A systematic search was performed in three scientific databases—the Cochrane Central Register of Controlled Trials (CENTRAL), Embase and Medline (via PubMed)—for studies published up to 1 December 2024. The following search key was used in all databases: (remote monitor* OR home monitor OR telemonitor* OR “remote” OR “implantable hemodynamic monitor*”) AND (“implantable device” OR CIED OR implantable cardioverter defibrillator OR cardiac resynchronization therapy OR ICD OR CRT). No restrictions (year, language, etc.) were made.

### 2.2. Selection and Eligibility Criteria

The search results from the three selected databases were imported into a citation management software (EndNote X9, Clarivate Analytics, Philadelphia, PA, USA) for systematic selection. Following the removal of both automatic and manually identified duplicates, the study selection was conducted in two stages by two independent reviewers (B.V. and B.K.). Initially, selection was based on the title and abstract review, followed by a full-text review in the second stage. After each stage, the level of agreement between reviewers was assessed using Cohen’s Kappa statistic to evaluate the consistency of the selection process. Any disagreements regarding the eligibility of a study were resolved through consultation with a third reviewer (A.K.). The inclusion criteria were limited to randomized controlled trials that compared remote monitoring with standard in-clinic follow-up in HF patients, specifically focusing on outcomes such as all-cause mortality, HF hospitalization and cardiovascular-related hospitalizations. Studies that were non-randomized were excluded from this analysis.

### 2.3. Data Extraction

A structured data collection form was employed to extract data from the selected studies for quality evaluation and evidence synthesis. The extracted data included details about the study characteristics, participant demographics and outcomes (for dichotomous variables, the number of patients experiencing each outcome; for continuous variables, the mean with standard deviation or median with interquartile range). Data extraction was performed independently by two authors (B.V. and B.K.), and any discrepancies were resolved through mutual agreement.

### 2.4. Risk of Bias Assessment

Two independent reviewers (B.V. and B.K.) evaluated the risk of bias in the included studies using the “Revised Cochrane Risk-of-Bias Tool for randomized trials” (RoB 2). Any disagreements regarding the risk of bias assessments were addressed and resolved with the involvement of a third reviewer (A.K.), as detailed in Supplementary Materials Figures S2–S8 [12].

### 2.5. GRADE

**Figure 1:**
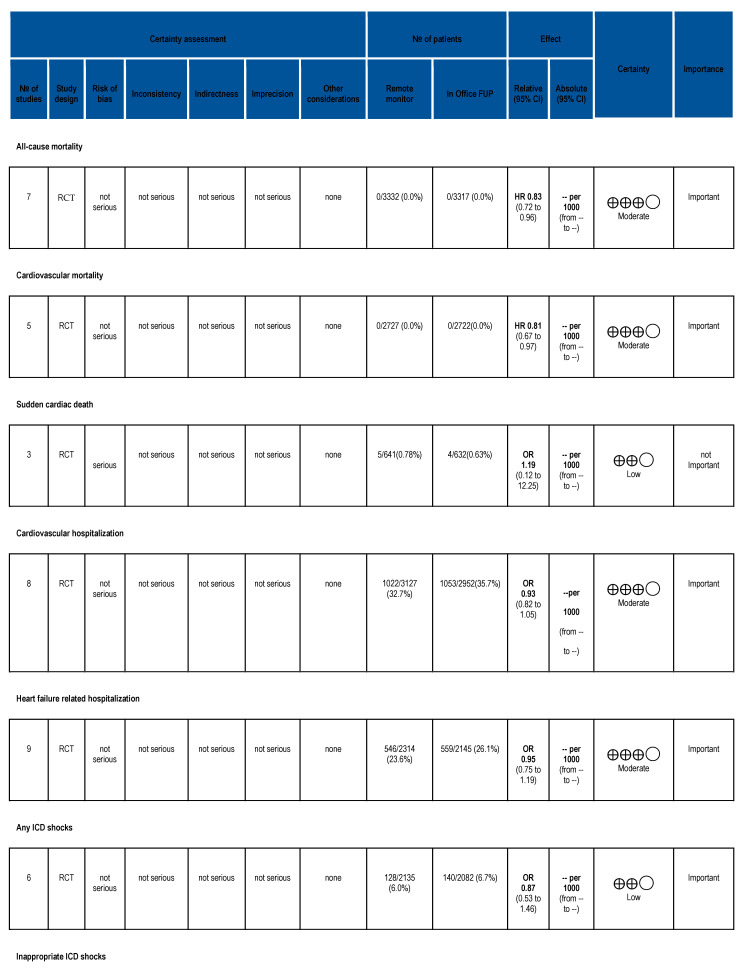

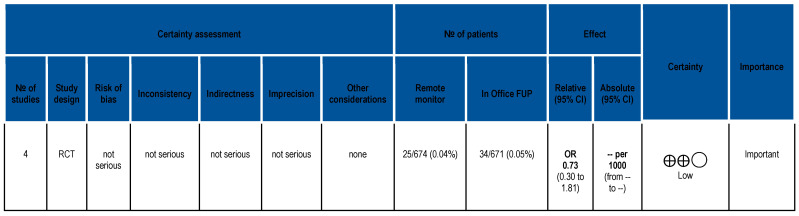
GRADE.

Two reviewers (B.V. and B.K.) performed the grading of trials and outcomes (Grading of Recommendations, Assessment, Development, and Evaluations, GRADE). Disagreements between the two reviewers were resolved by a third reviewer (A. K.). The grading was performed using GRADEpro (https://www.gradepro.org/ (accessed on is 21 January 2022)) Guideline Development Tool [Software]. McMaster University and Evidence Prime, 2024. (Figure 1) [13].

### 2.6. Statistical Analysis

The effect size measures used were odds ratios (ORs) and hazard ratios (HRs), each with 95% confidence intervals (CIs). Due to the higher robustness of hazard ratios, where both event numbers and hazard ratios were available from enough studies for pooling, hazard ratios were prioritized. For outcomes with too few studies for an HR meta-analysis, the OR was calculated. The pooled OR based on raw data was calculated using the Mantel–Haenszel method. The exact Mantel–Haenszel method, without continuity correction, was employed to handle zero-cell counts [14]. To ensure more conservative estimates, the pooled CI was modified using the Hartung–Knapp method when applicable [15]. The 95% CI of the random effects model describes the precision of the pooled estimate across the included studies, indicating a statistically significant average effect if it does not cross 1.0.

The prediction interval, however, considers between-study heterogeneity and predicts the likely range of effect sizes in future similar studies.

For the reported HRs, the HRs were pooled using the inverse variance weighting method. The restricted maximum-likelihood (REML) estimator was used to account for between-study variability, and the Q-profile method was applied for the confidence interval [16]. Both ORs and HRs were pooled using random-effects meta-analysis models to account for the variability across studies. Heterogeneity among the studies was assessed using the Higgins and Thompson I 2 statistic, which quantifies the proportion of variation in effect estimates due to heterogeneity rather than sampling error [17]. Small study bias was assessed by a visual inspection of funnel plots [18].

All analyses were performed using random-effects meta-analysis models in R version 4.2.1 with the following packages: dmetar (Harrer et al., 2019) [15] and metafor (Viechtbauer, 2010) [16].

## 3. Results

**Figure 2:**
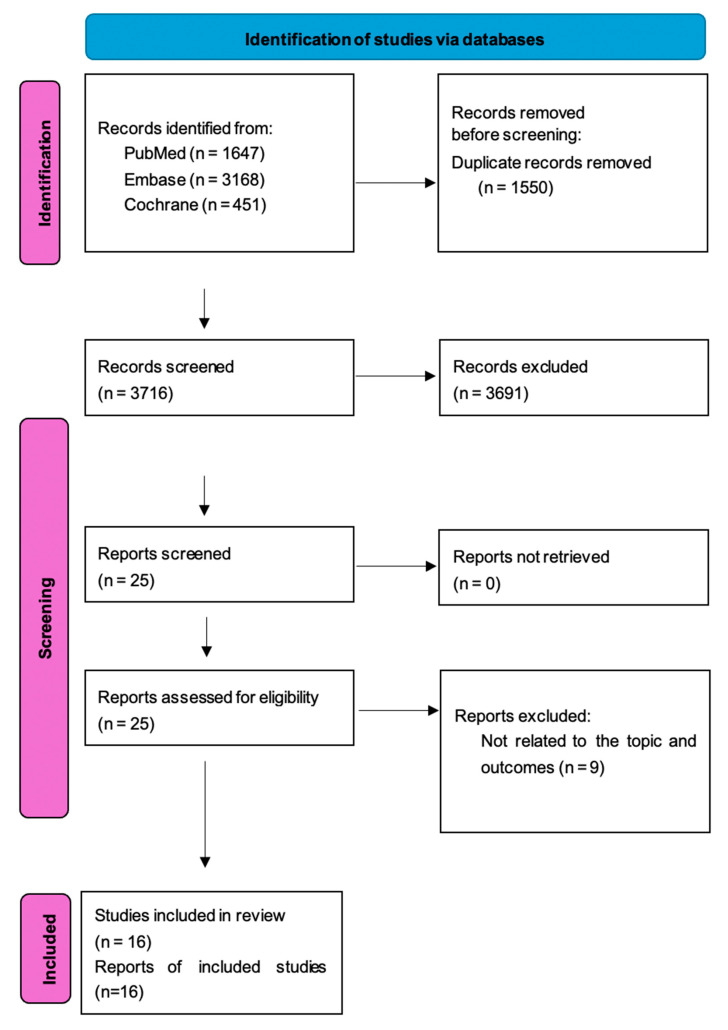
PRISMA flow chart of search for publications.

A total of 16 studies were selected for the current analysis, comprising 11,232 HF patients, including 6288 patients followed up remotely and 4944 patients followed up by in-person visits. (Figure 2, Table 1a). All articles were randomized controlled trials. Additional information regarding the following aspects is provided in Supplementary Materials Table S1: study design distribution of CRT-D and ICD patients in the RTM and IPV groups, and follow-up schedules. Moreover, the table includes a comparison of inclusion and exclusion criteria across studies to assess population comparability and potential heterogeneity. Details concerning remote monitoring protocols, including system components, timing (baseline and/or follow-up), and evaluation procedures, are also outlined. Additionally, information related to the adjudication method subgroup effect is summarized where available. Although different studies used different algorithms and parameters, the main differences were daily and non-daily reporting on the RTM arm and regularity of in-office visits. We undertook a subgroup analysis based on these parameters, and no statistically significant difference was found between RTM vs. in-person visit regarding daily and non-daily reporting, regularity of in-office visits (3, 6, 12 months) or other intervals (or thoracic impedance (TI) monitoring vs. no TI monitoring). We report our results in the Supplementary Materials.

### 3.1. Baseline Clinical Characteristics of Patients

The reported mean age in the trials was between 60 and 70 years. All articles showed a greater proportion of males, comprising over 70% in total. The mean LVEF was 25–30%, and a majority of patients (approx. 60%) had ischemic etiology HF. Approximately 90% of the patients used ACE inhibitors/ARB, BB treatment and diuretics treatment. The presence of NYHA III-IV patients was heterogeneous, accounting for 5–81% of the investigated patients (Table 1a,b).

### 3.2. Mode of Death

#### 3.2.1. All-Cause Mortality

**Figure 3:**
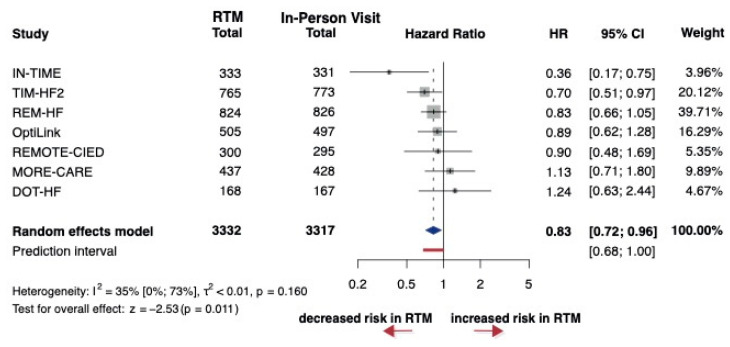
Forest plot of all-cause mortality. Hazard ratios (HRs) and 95% confidence intervals (CI) are shown for each study. The blue diamond represents the pooled HR, and the red bar shows the prediction interval. HR < 1 favors RTM [27,28].

Regarding all-cause mortality, 15 studies were found comparing RTM to in-person visits; however, only 7 studies reported HRs, which were included in the analysis, encompassing a total of 6649 patients. The HR was 0.83 between RTM vs. in-person visits groups. The 95% CI of HR was 0.72 to 0.96, which showed a statistically significant difference between RTM vs. in-person visits (*p* = 0.01) (Figure 3). Overall, a majority of the studies demonstrated some concerns, particularly regarding missing data and selective reporting (Supplementary Materials Figure S2).

#### 3.2.2. Cardiovascular Mortality

**Figure 4:**
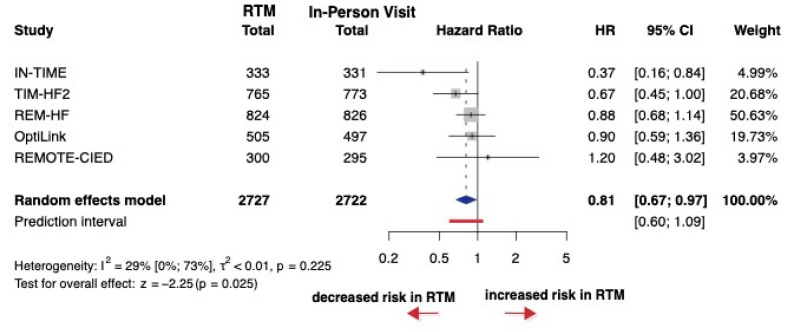
Forest plot of cardiovascular mortality. Hazard ratios (HRs) and 95% confidence intervals (CI) are shown for each study. The blue diamond represents the pooled HR, and the red bar shows the prediction interval. HR < 1 favors RTM [27,28].

For cardiovascular mortality, 11 studies were identified comparing RTM to in-person visits; however, only 5 studies reported HRs, which were included in the analysis, encompassing a total of 5449 patients. The HR was 0.81 between RTM and in-person visits. The 95% CI of HR was 0.67 to 0.97, which represents a significant difference between RTM and in-person visits (*p* = 0.025) (Figure 4). The overall risk of bias varied slightly across studies, with most showing some concerns, particularly regarding the handling of missing data and the selection of reported outcomes. (Supplementary Materials Figure S3).

#### 3.2.3. Sudden Cardiac Death

Only three trials were included for the investigation of sudden cardiac death, encompassing a total of 1273 patients. These studies did not report HRs. The OR (the pooled effect size) was 1.19 between RTM and in-person visits. The 95% CI of OR was 0.12 to 12.25, which showed no benefit of RTM compared to in-person visits (Supplementary Materials Figure S1). The overall assessment indicated that while two of the studies presented some concerns (Hindricks et al. [10] and Luthje et al. [24]), the Guédon-Moreau et al. [23] study had a low risk of bias. These assessments are critical for interpreting the validity and reliability of findings related to sudden cardiac death (Supplementary Materials Figure S4).

### 3.3. Hospitalization

#### 3.3.1. Cardiovascular Hospitalization

**Figure 5:**
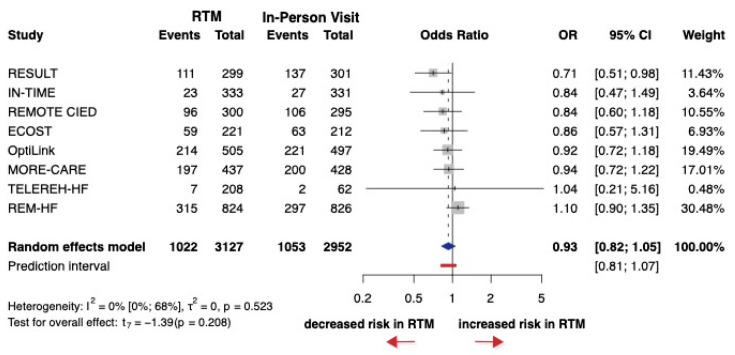
Forest plot of cardiovascular hospitalization. Odds ratios (ORs) and 95% confidence intervals (CI) are shown for each study. The blue diamond represents the pooled OR, and the red bar shows the prediction interval. OR < 1 favors RTM.

A total of eight studies were selected for analyses, encompassing a total of 6079 patients, with only four studies reporting HRs. The OR (the pooled effect size) was 0.93 between RTM and in-person visit groups. The 95% CI of OR was 0.82 to 1.05, which showed no significant difference between RTM and in-person visits (*p* = 0.208) (Figure 5). For cardiovascular hospitalization, most studies had an overall low risk of bias, with some showing specific areas of concern, particularly in allocation concealment and missing data (Supplementary Materials Figure S5).

#### 3.3.2. Heart-Failure-Related Hospitalization

**Figure 6:**
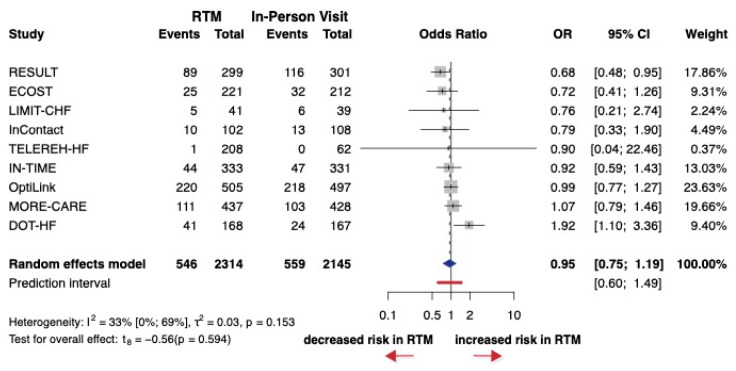
Forest plot of heart-failure-related hospitalization. Odds ratios (ORs) and 95% confidence intervals (CI) are shown for each study. The blue diamond represents the pooled OR, and the red bar shows the prediction interval. OR < 1 favors RTM.

A total of nine studies, encompassing 4289 patients, were selected for analysis. Eight of the nine studies did not report HRs. The OR (the pooled effect size) was 0.95 between RTM and in-person visit groups. The 95% CI of OR was 0.75 to 1.2, clarifying that there was no significant difference in the effect of the two groups (*p* = 0.594) (Figure 6). The overall risk of bias for most studies was low, although some studies showed concerns primarily due to missing data, deviations from protocol or inconsistencies in reporting (Supplementary Materials Figure S6).

### 3.4. ICD Therapies

#### 3.4.1. Any ICD Shocks

**Figure 7:**
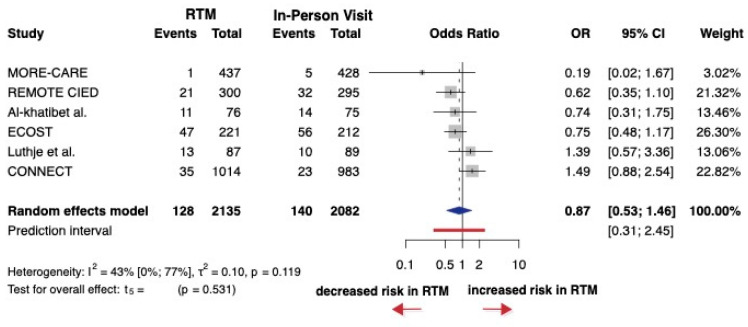
Forest plot of any ICD shocks. Odds ratios (ORs) and 95% confidence intervals (CI) are shown for each study. The blue diamond represents the pooled OR, and the red bar shows the prediction interval. OR < 1 favors RTM.

A total of six studies were selected for analyses, covering a total of 4217 patients. Five of the six studies did not report hazard ratios. The OR (the pooled effect size) was 0.87 between RTM and in-person visit groups. The 95% confidence interval of OR was 0.53 to 1.46, which demonstrates that RTM did not significantly decrease the rate of ICD shocks compared with in-person visits (*p* = 0.531) (Figure 7). For this endpoint, most studies showed some concerns, especially related to missing data, deviations from intended interventions, and potential selective reporting (Supplementary Materials Figure S7).

#### 3.4.2. Inappropriate ICD Shocks

**Figure 8:**
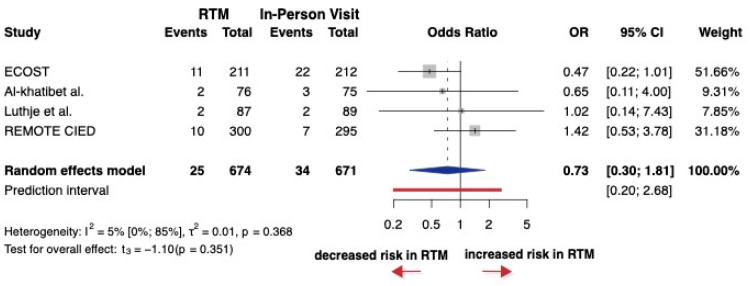
Forest plot of inappropriate ICD shocks. Odds ratios (ORs) and 95% confidence intervals (CI) are shown for each study. The blue diamond represents the pooled OR, and the red bar shows the prediction interval. OR < 1 favors RTM [24].

A total of four studies were selected for analyses, covering a total of 1345 patients. Three of the four studies did not report hazard ratios. The OR (the pooled effect size) was 0.73 between RTM and in-person visits. The 95% confidence interval of OR was 0.30 to 1.81, which means RTM did not have an effect on the rate of inappropriate therapy (*p* = 0.351) (Figure 8). These studies generally showed some concerns, particularly related to missing data and issues within the randomization process (Supplementary Materials Figure S8). These findings may be caused by the differences in alert action thresholds, follow-up protocols or patient selection.

#### 3.4.3. Subgroup Analysis

As different RTM systems operate through distinct mechanisms, this may substantially influence the outcomes and hinder direct comparisons. To address this limitation, we performed a subgroup analysis based on the most significant differences, like the daily or non-daily alert system or the frequency of in-office visits. Follow-up is most commonly conducted at 3, 6 and 12 months, although the timing varies between studies. No statistically significant differences were found between RTM and in-person visits in terms of daily versus non-daily reporting, the regularity of in-office visits (at 3, 6 or 12 months), or other follow-up intervals (Supplementary Figures S10 and S11).

#### 3.4.4. Sensitivity Analysis

To evaluate the robustness of the mortality outcomes, we performed a sensitivity analysis excluding studies deemed of low quality. Our findings indicate no statistically significant difference between studies with low risk of bias and those with some concerns, based on our risk of bias assessment (Supplementary Figure S12).

### 3.5. Risk of Bias Assessment and GRADE

Following the assessment of risk of bias, all of the enrolled studies showed low or moderate risk of bias (Supplementary Materials, Figures S2–S8). Using the GRADE approach to grade the evidence in systematic reviews, moderate certainty was established (Figure 1).

## 4. Discussion

This systematic review and meta-analysis investigated the impact of remote telemedical management (device-related or non-related) in cardiac implantable electronic device (CIED) patients with heart failure (HF). Our meta-analysis showed a substantial benefit in remote monitoring, which was associated with an overall reduction in all-cause mortality and cardiovascular-related mortality. This aspect holds notable importance as within the care of HF, despite the widespread adoption of guideline-recommended therapies, the mortality rates among HF patients continue to persist at higher levels. As a result, this ailment remains a significant healthcare concern, with hospitalization accounting for the majority of HF expenses [34].

Telemedicine or remote monitoring in HF encompasses various approaches, from computer-based systems to those supervised by healthcare professionals [35]. To ensure a precise understanding of clinical trials, it is crucial to standardize and appropriately classify telemedical systems.

Over the past decade, numerous trials examining telehealth interventions with different modalities and outcomes in HF patients have been published. Notable trials such as DOT-HF, LIMIT-CHF, OPTILINK and a study by Luthje et al. have focused on remote patient follow-ups by monitoring thoracic impedance and fluid accumulation [11,21,24,26,27]. Based on their outcomes, no discernible mortality advantage was observed between RTM and in-person visits.

The RESULT, REMOTE-CIED and MORE-CARE studies used complex methodologies for remote patient monitoring [25,30,32]. In the RESULT trial, a notable discrepancy emerged between the RTM group and in-person visits group in terms of cardiovascular and heart failure-related hospitalization [30]. The MORE-CARE study yielded neutral findings across all outcomes, while the REMOTE-CIED study found a benefit in appropriate ICD shocks in the RTM group [25,32].

To date, the only randomized controlled trial that has demonstrated a significant positive impact on a primary composite clinical score—encompassing all-cause mortality, hospitalization for HF, changes in NYHA class, and alterations in patient global self-assessment—and mortality alone was the IN-TIME study [10,36]. The benefits of telemonitoring in this cohort may be associated with early detection and treatment of atrial and ventricular tachyarrhythmias, prompt identification of suboptimal device function, and patient interviews that occasionally revealed a worsening of HF or intolerance to HF medications.

RTM and interview-based therapy modifications can enable the safe reduction of in-person visits, thereby contributing to the prevention of hospitalization. Even though the threshold values for the alerts performed well in the study, they were empirically used, lacking scientific support or optimization. An ongoing trial is attempting to clarify this question (NCT0617308).

Despite the TIM-HF2 trial consisting of only 50% of patients with Cardiovascular Implantable Electronic Devices (CIEDs), the implemented remote management system proved valuable for monitoring HF patients [33]. Koehler et al. [37] selectively incorporated individuals at a heightened risk for HF hospitalization, deliberately excluding those with major depression. This strategic decision stemmed from their prior TIM-HF study, which encountered challenges in elucidating distinctions between remote monitoring and standard care, largely attributed to a substantial prevalence of major depression within the participant cohort [37]. These findings may imply that a holistic integration of RTM, incorporating a blend of devices and additional measures, along with collaboration between arrhythmia and HF physicians, could prove to be a more efficacious strategy within the HF population compared to a simplistic device-based approach.

Based on the randomized and observational trials released in previous years on the topic, a substantial number of meta-analyses and systematic reviews were performed [38,39]. The most recent was published in May 2023, and evaluated all articles related to invasive and non-invasive remote monitoring systems [4]. Scholte et al. have found that the use of remote monitoring systems can reduce all-cause mortality and first- and overall HF hospitalization [4]. However they concluded that the wide variety of telemonitoring system methods necessitates further research to establish standardized protocols for effective remote monitoring [4]. Our findings have led us to the same conclusion.

Our main focus was on complex telemonitoring support and the invasive CIED-based method of RTM. From these two groups of patients, we obtained similar mortality results to those described by Scholte et al. [4]. However, we did not find any differences in total cardiovascular and HF hospitalizations. The reason for our findings could be that in CIED remote monitoring systems, alert parameters and cut-off values are set up empirically, making it challenging to determine when a patient should be sent to the hospital or emergency unit to prevent HF hospitalization due to drug changes. Therefore, these parameters remain uncertain and warrant investigation through well-designed clinical trials.

## 5. Conclusions

In summary, our comprehensive meta-analysis revealed an association between telemedical management and reduced all-cause and cardiovascular mortality as compared to traditional in-patient visits in patients with HF. It is imperative that future research endeavors investigate clinically pertinent indicators that can reliably predict hospital admissions, arrhythmic events or HF hospitalization.

## 6. Clinical Implication

Drawing on the findings from our systematic review and meta-analysis, we are optimistic that the effectiveness of remote telemedical management can be further enhanced via its routine implementation alongside the adoption of standardized protocols using fast reactions to alerts, with the potential for improving overall outcomes for patients.

Moreover, translating scientific findings into clinical practice is of paramount importance and has been emphasized as a top priority by the Academia Europaea [40,41].

## 7. Future Perspective

Future research should focus on developing and validating standardized RTM protocols and alert thresholds to improve reproducibility across studies. Establishing consensus guidelines for RTM implementation in clinical settings will enhance decision-making and optimize patient outcomes. By addressing these gaps, future studies can provide clearer guidance on the integration of RTM into cardiovascular care.

## 8. Strengths and Limitations

This systematic review and meta-analysis has two major strengths. First, we were able to evaluate most of the important hard endpoints related to CIED systems, as opposed to earlier meta-analyses. In our opinion, this manner of analyzing the data is crucial since these outcomes have different implications and economic impacts. Second, we were able to show statistical significance between RTM and in-person visits regarding all-cause mortality and cardiovascular mortality, which was a limitation of previous meta-analyses.

However, this systematic review and meta-analysis also has some limitations. First, various types of CIEDs were not included in the representation, and the studies did not encompass patients with single-chamber or dual-chamber pacemakers, individuals with HF with preserved ejection fraction (HFpEF) or those with subcutaneous, leadless or conduction system pacing. Second, the implementation of RTM exhibited considerable diversity across the trials, with the studies utilizing different remote monitoring devices and parameters. Third, the included TIM-HF2 trial encompassed only 50% of patients with Cardiovascular Implantable Electronic Devices (CIEDs).

Another limitation is that some articles did not report hazard ratios, which reduced the interpretability of our analysis, and as our meta-analysis is not an individual patient data meta-analysis, this is an inherent limitation.

The included trials varied significantly in follow-up duration, ranging from 12 to 34 months, and hazard ratios were calculated based on the total follow-up time of each study, potentially introducing inconsistencies in the assessment of long-term outcomes, starting from the date of randomization. These factors should be considered when interpreting the results and their applicability to broader clinical practice. Moreover, since our primary objective was not to evaluate pharmacological therapies for heart failure, we did not investigate the potential interactions between the evolution of medical therapy, device-based treatments and remote monitoring.

## Figures and Tables

**Table 1:** Characteristics of enrolled studies, and baseline clinical characteristics of patients.

(a)																
Author, Year	Trial Name	Number of Patients		CIED		Follow- Up (Month)		Age (Mean ± SD), Median (IQR)			Male (%)			Mean LVEF (%) (Mean ± SD) Median (IQR)		
		RTM	IPV	CRT-D	ICD			RTM	IPV		RTM		IPV	RTM		IPV
Varma 2010 [19]	TRUST	908	431	0	1339	15		63.3 ± 12.8	64.0 ± 12.1		72		73.1	29.0 ± 10.7		28.5 ± 9.8
Al-khatib 2010 [20]	Pilot study	76	75	28	123	12		63 (54–72)	63 (54–70)		72		73	25 (20–35)		28 (20–35)
Veldhuisen 2011 [21]	DOT-HF	168	167	274	61	15		64 ± 10	64 ± 10		87		85	25 ± 7		25 ± 7
Crossley 2011 [22]	CONNECT	1014	983	757	1240	15		65.2 ± 12.4	64.9 ± 11.9		70.5		71.7	28.6 ± 10		29.2 ± 10.3
Guedon-Moreau 2012 [23]	ECOST	221	212	78	232	27		62.0 ± 13.0	61.2 ± 12.0		87.3		89.2	34.7 ± 13.0		35.1 ± 13.6
Hindricks 2014 [10]	IN-TIME	333	331	274	390	12		65.3 ± 9.3	65.8 ± 9.6		82.3		79.2	26 ± 6		26 ± 7
Luthje 2015 [24]	Pilot study	87	89	88	88	15		66.0 ± 12.0	65.9 ± 12.1		80.5		74.2	32.7 ± 11.4		31.1 ± 10.2
Boriani 2016 [25]	MORE -CARE	437	428	865	0	24		66 ± 11	67 ± 10		78.8		73.1	27.3 ± 6.6		27.4 ± 6.0
Domenichini 2016 [26]	LIMIT-CHF	41	39	53	27	12		69.5 ± 11.0	66.3 ± 11.9		98		90	28.9 ± 8.4		27.6 ± 7.5
Böhm 2016 [27]	OptiLink	505	497	627	375	23		66.1 ± 10.1	66.4 ± 10.7		77.2		82.3	26.7 ± 6.1		26.7 ± 6.1
Morgan 2017 [28]	REM-HF	824	826	1099	551	34		69.5 ± 10.31	69.5 ± 10.04		85.8		85.7	29.9 ± 10.24		30.0 ± 9.81
Hansen 2018 [29]	INCONTACT	102	108	68	142	13		62.5 ± 12.2	65.1 ± 10.1		83.3		81.2	28.2 ± 7.1		28.3 ± 8.9
Tajstra 2020 [30]	RESULT	299	301	249	351	12		64 ± 13	64 ± 12		81.6		80.7	27 ± 10		26 ± 10
Pluta 2020 [31]	TELEREH-HF	208	62	108	162	2.25		61.3± 11	62.2± 9		90.9		95.2	29.3± 6.9		27.9± 6.6
Chiu 2022 [32]	REMOTE-CIED	300	295	230	575	24		66 (58–73)	65 (59–73)		78		81	27 (21–31)		27 (21–31)
Koehler 2018 [33]	TIM-HF2	765	773	240	456	12		70 ± 11	70 ± 10		70		69	-		-
**(b)**																
**Author, year**	**Ischemic** **(%)**		**NYHA** **class (%)** **(III, IV)**		**ACEi/ARB** **(%)**			**Beta** **blocker** **(%)**		**MRA (%)**				**Diuretic** **(%)**		
	RTM	IPV	RTM	IPV	RTM		IPV	RTM	IPV	RTM		IPV		RTM	IPV	
Varma 2010 [19]	64.8	71.7	30.1	31.1	50.5		55.9	79.6	76.3	-		-		-	-	
Al-khatib 2010 [20]	66	67	5	0	85		77	88	89	-		-		63	71	
Veldhuisen 2011 [21]	52	60	39	36	83		87	92	92	-		-		92	87	
Crossley 2011 [22]	63.3	61.5	50	49	-		-	-	-	-		-		-	-	
Guedon-Moreau 2012 [ 23]	64.7	66.5	6.4	11.8	-		-	-	-	-		-		-	-	
Hindricks 2014 [10]	70	68	54.8	59.2	92.2		86.4	91.3	91.8	-		-		95.2	91.5	
Luthje 2015 [24]	58.6	43.8	37.9	47.7	-		-	-	-	-				-	-	
Boriani 2016 [25]	42.8	45.3	62.9	61.1	82.4		79	89.1	88.3	31.5		34		91.7	91.2	
Domenichini 2016 [26]	78	72	-	-	95		97	90	90	66		46		81	85	
Böhm 2016 [27]	54.3	54.5	80.4	80.9	91.3		94	94.5	92.4	68.9		69.6		95	95	
Morgan 2017 [28]	68.7	67	29	32	91		91.3	90.9	90.3	52.2		52.7		77.1	76.4	
Hansen 2018 [29]	56.9	61.1	44.1	41.7	97		94.4	-	-	53.5		56.5		85.3	82.4	
Tajstra 2020 [30]	63.2	64.8	17.9	24.2	96.4		93	90	91.4	95.9		98.9		89	90.7	
Pluta 2020 [31]	64.9	56.4	17.3	30.6	95.7		95.2	97.6	98.4	85.6		96.8		78.4	82.3	
Chiu 2022 [32]	53	60	33	34	89		88	82	85	59		65		72	73	
Koehler 2018 [33]	39	42	47	47	82		83	92	92	58		52		94	93	

RTM: Remote telemedical management, IPV: in-person visit.

## Supplementary Materials

The following supporting information can be downloaded at https://www.mdpi.com/article/10.3390/jcm14124278/s1. Figure S1: Forest plot of sudden cardiac death; Figure S2: Revised Cochrane Risk-of-Bias Tool for randomized trials (RoB 2) in trials evaluating all-cause mortality; Figure S3: Revised Cochrane Risk-of-Bias Tool for randomized trials (RoB 2) in trials evaluating cardiovascular mortality; Figure S4. Revised Cochrane Risk-of-Bias Tool for randomized trials (RoB 2) in trials evaluating sudden cardiac death; Figure S5: Revised Cochrane Risk-of-Bias Tool for randomized trials (RoB 2) in trials evaluating cardiovascular hospitalization; Figure S6: Revised Cochrane Risk-of-Bias Tool for randomized trials (RoB 2) in trials evaluating heart failure hospitalization; Figure S7: Revised Cochrane Risk-of-Bias Tool for randomized trials (RoB 2) in trials evaluating any ICD shocks; Figure S8: Revised Cochrane Risk-of-Bias Tool for randomized trials (RoB 2) in trials evaluating inappropriate ICD shocks; Figure S9: PRISMA checklist; Figure S10: Forest plot of all-cause mortality for the subgroup analysis of daily and non-daily reporting on the RTM; Figure S11: Forest plot of all-cause mortality for the subgroup analysis of regularity of in-office visits (3,6,12 months) on the RTM; Figure S12: Forest plot of all-cause mortality for the sensitivity analysis based on Rob2 low and some concerns; Table S1: Additional information about the trials [42].

## Abbreviations

CI	Confidence interval
CIED	Cardiac implantable electronic devices
CRT	Cardiac resynchronization therapy
CV	Cardiovascular
HF	Heart failure
HFH	Heart failure hospitalizations
HFpEF	HF with preserved ejection fraction
HFrEF	HF with reduced ejection fraction
HR	Hazard ratio
ICD	Implantable cardioverter defibrillators
IPV	In-person visit
NYHA	The New York Heart Association
OR	Odds ratio
RCT	Randomized controlled trial
REML	Restricted maximum-likelihood
RTM	Remote telemedical management
STS	Structured telephone support
