# Supplementary material for: Enhancing Heart Failure Management: A Systematic Review and Meta-Analysis of Continuous Remote Telemedical Management vs. In-Person Visit in Patients with Cardiac Implantable Electronic Devices
Source: J Clin Med. 2025 Jun 16;14(12):4278. doi: 10.3390/jcm14124278 (PMC12194402; doi:10.3390/jcm14124278)
Supplement: Supplementary file 1 [file jcm-14-04278-s001.zip › jcm-3656386-supplementary.pdf]

Figure S1. Forest plot of sudden cardiac death

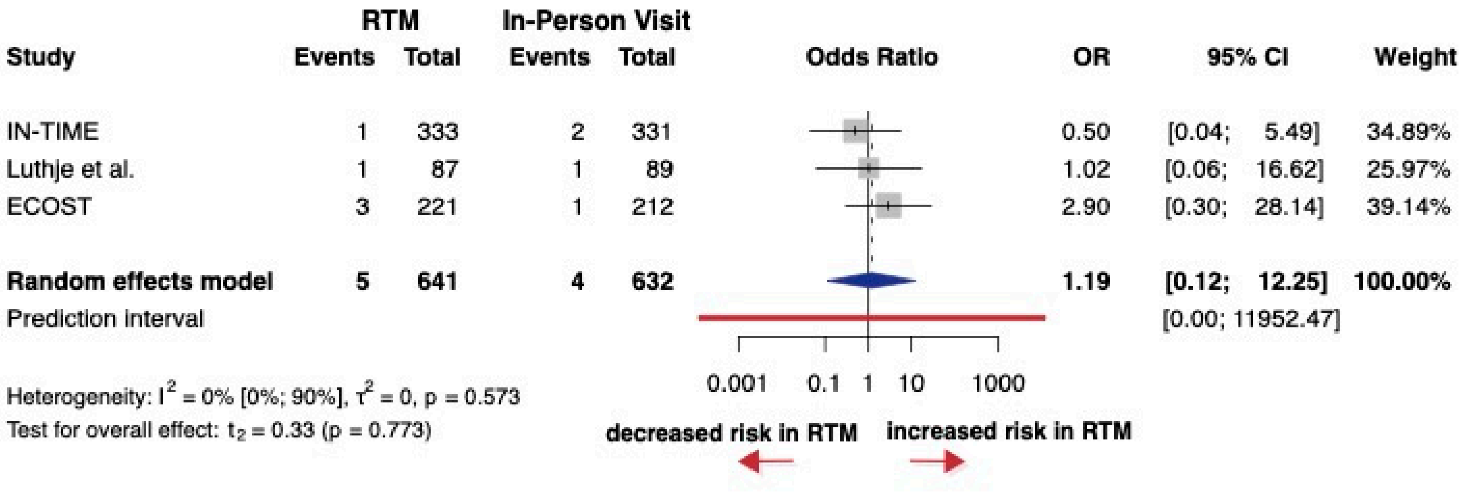

Figure S2. Revised Cochrane Risk-of-Bias Tool for randomized trials (RoB 2) in trials evaluating all-cause mortality

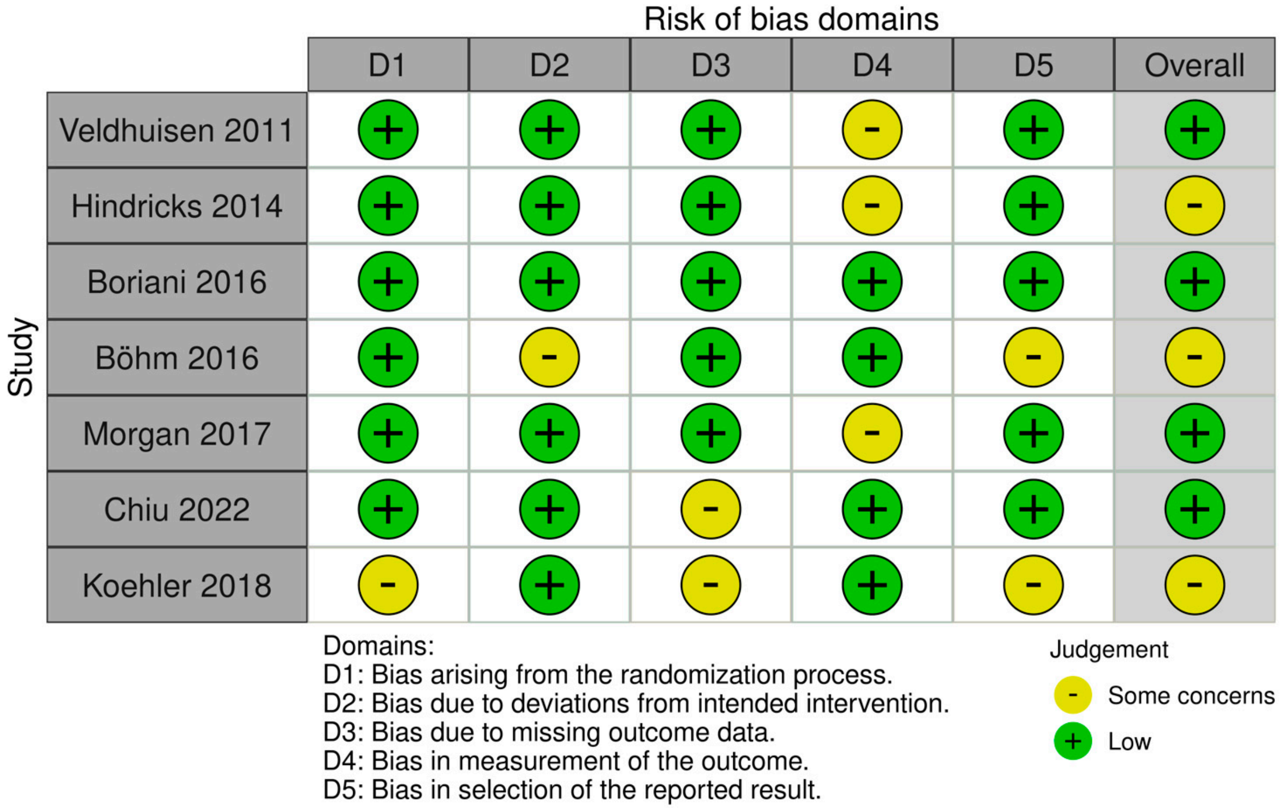

Figure S3. Revised Cochrane Risk-of-Bias Tool for randomized trials (RoB 2) in trials evaluating cardiovascular mortality

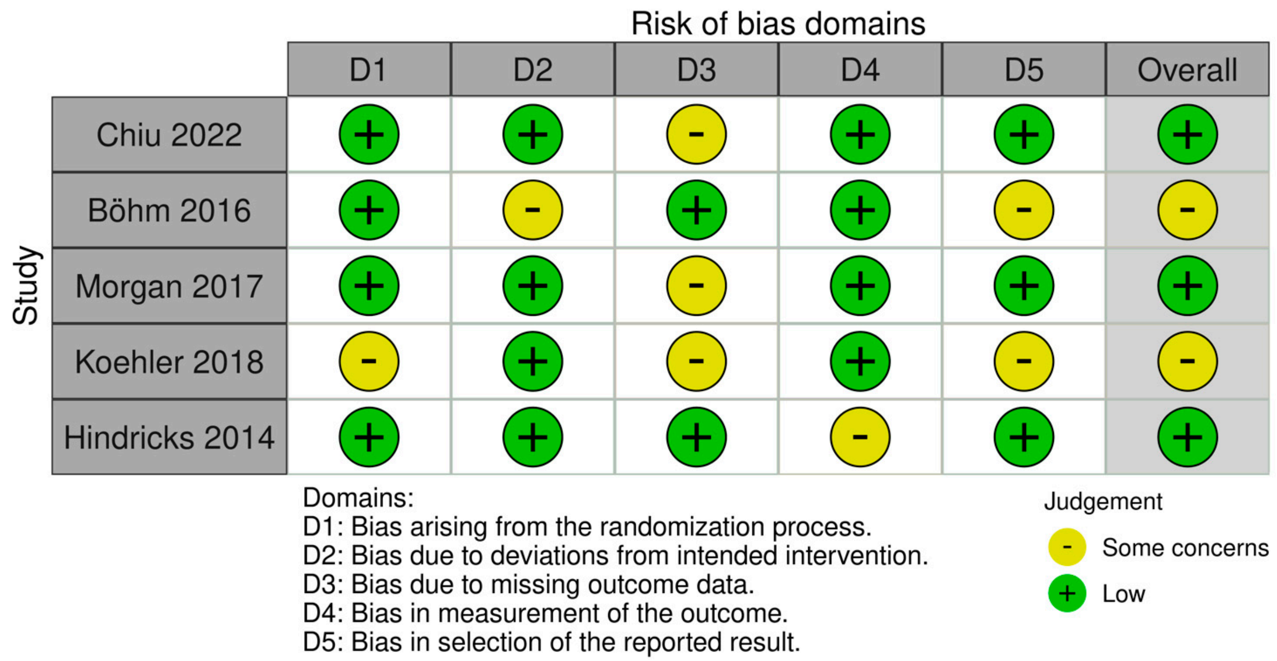

Figure S4. Revised Cochrane Risk-of-Bias Tool for randomized trials (RoB 2) in trials evaluating sudden cardiac death

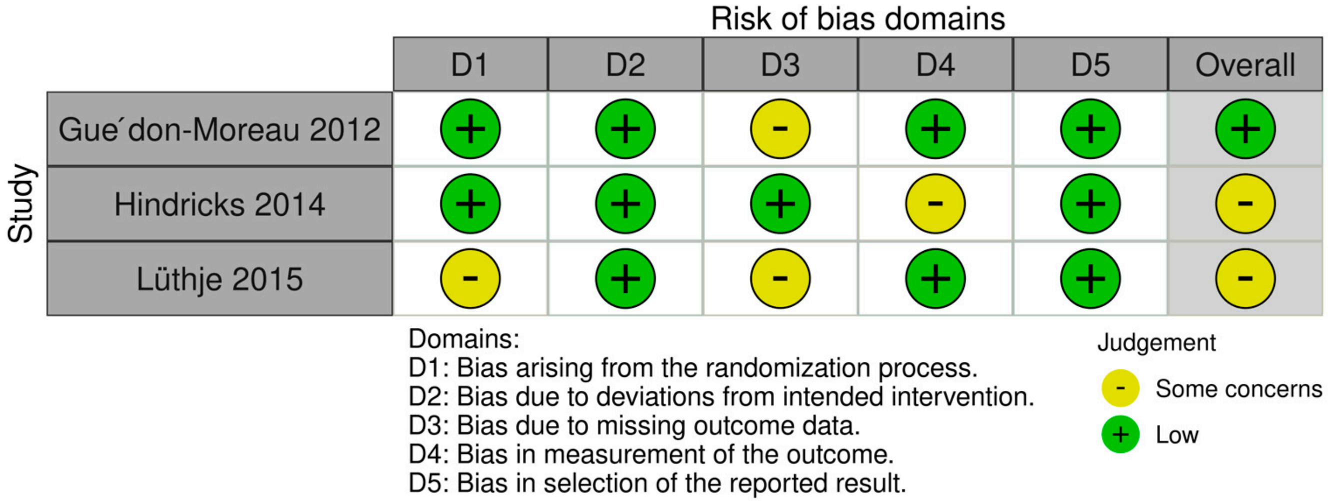

Figure S5. Revised Cochrane Risk-of-Bias Tool for randomized trials (RoB 2) in trials evaluating cardiovascular hospitalization

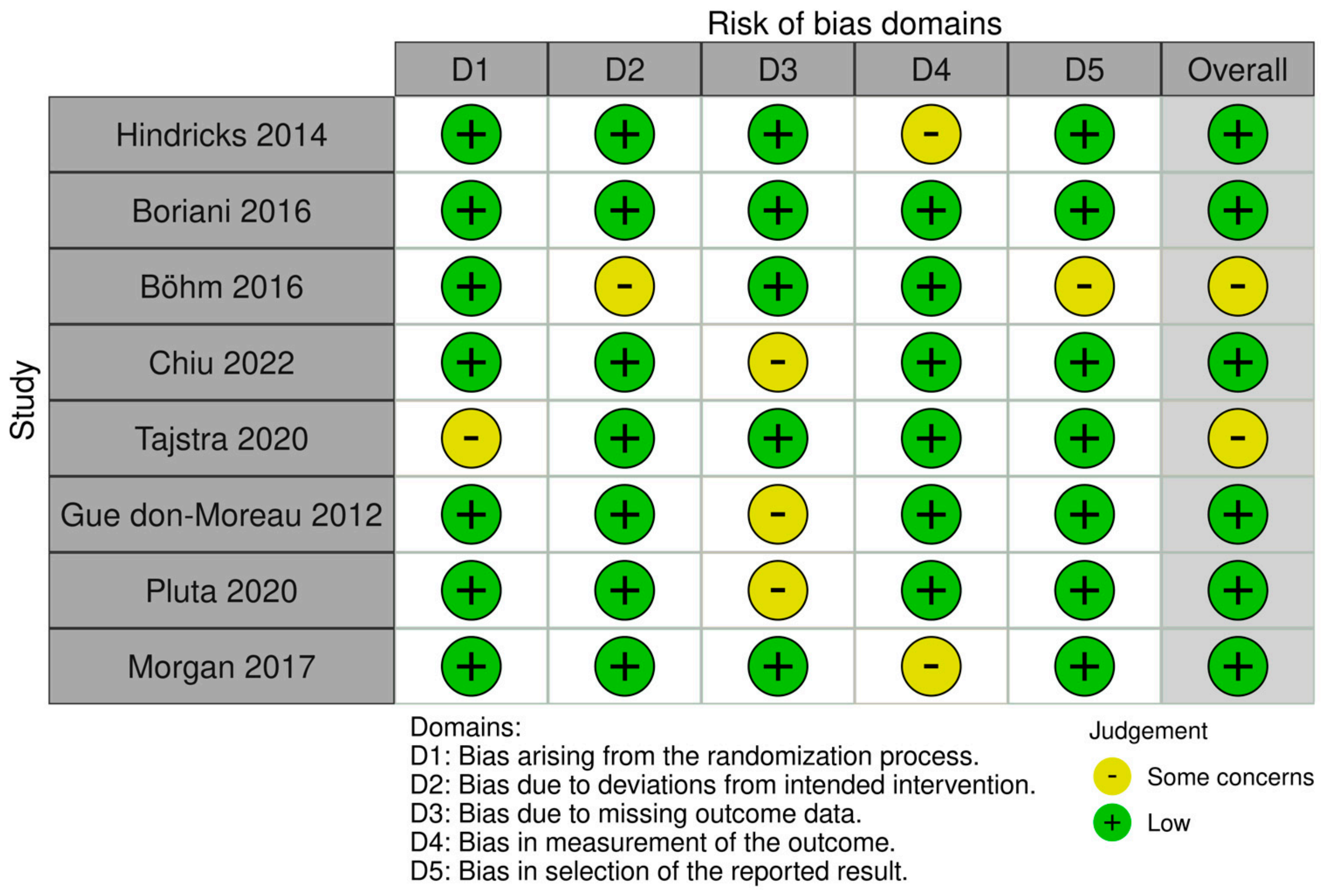

Figure S6. Revised Cochrane Risk-of-Bias Tool for randomized trials (RoB 2) in trials evaluating heart failure hospitalization

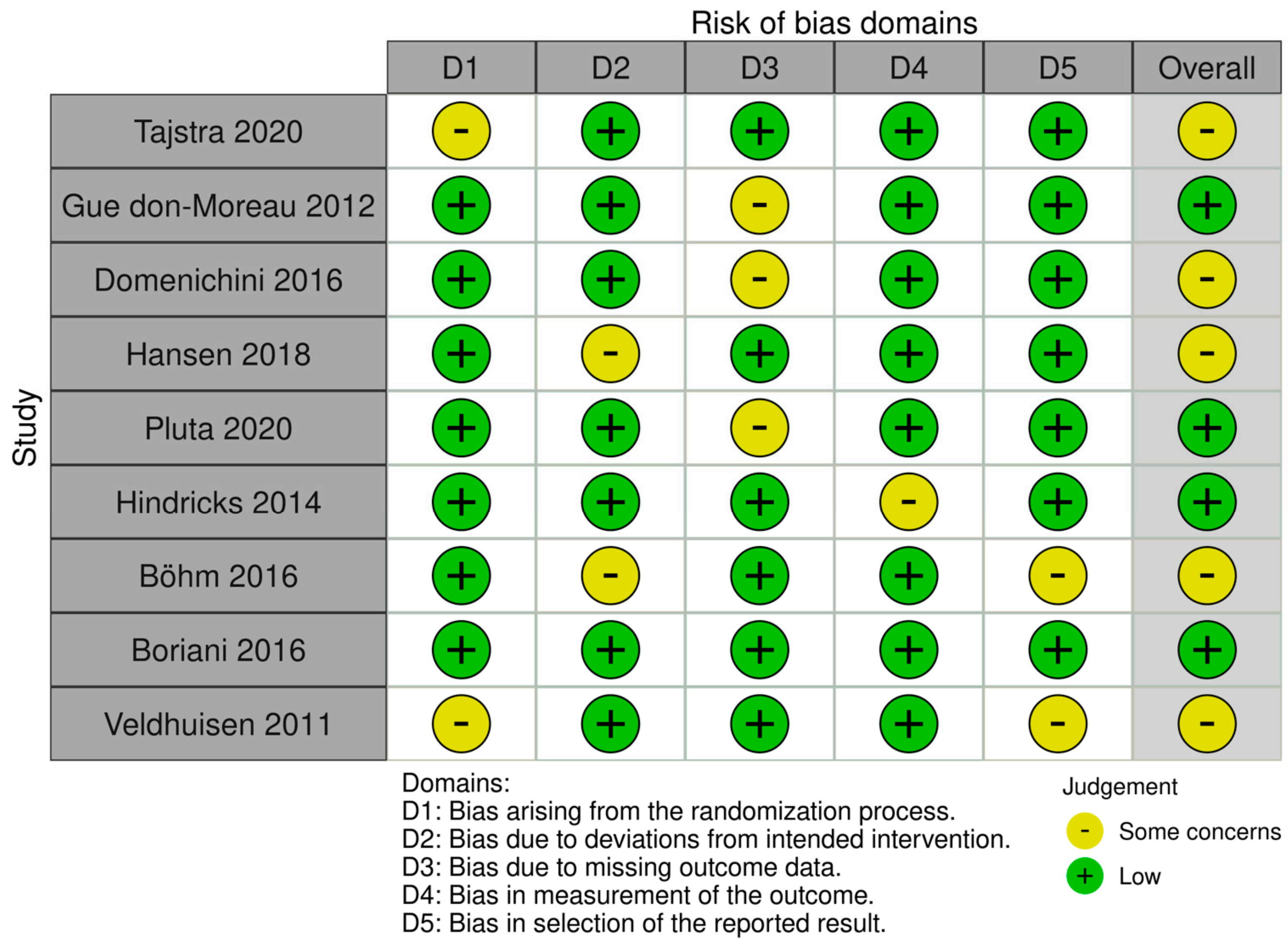

Figure S7. Revised Cochrane Risk-of-Bias Tool for randomized trials (RoB 2) in trials evaluating any ICD shocks

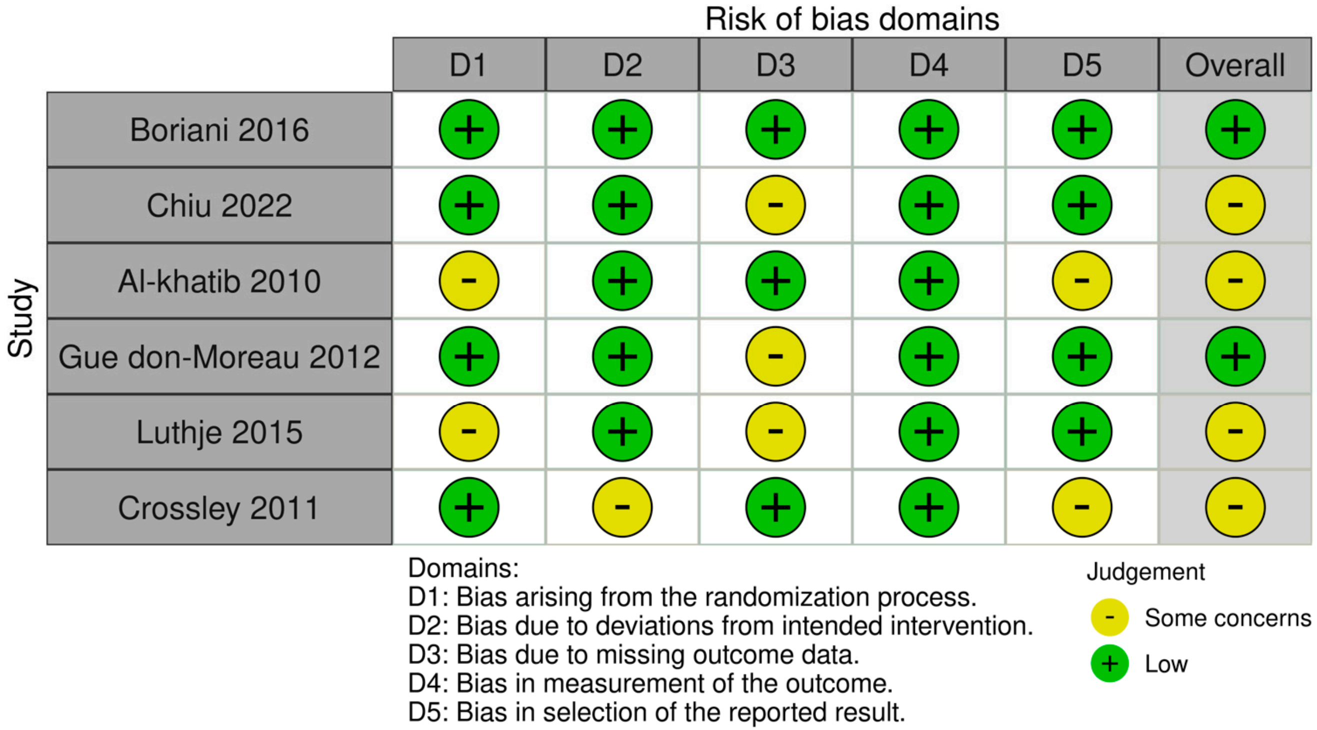

Figure S8. Revised Cochrane Risk-of-Bias Tool for randomized trials (RoB 2) in trials evaluating inappropriate ICD shocks

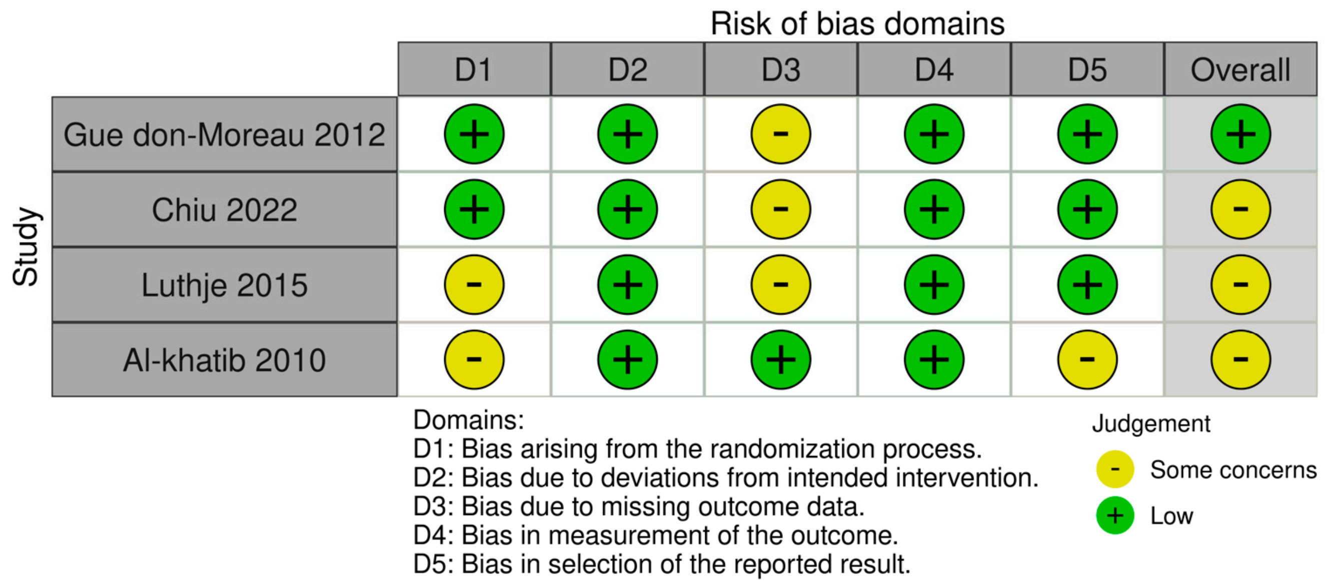

Figure S9. PRISMA checklist

Section and Topic	Item #	Checklist item	Location where item is reported
**TITLE**			
Title	1	Identify the report as a systematic review.	1
**ABSTRACT**			

Section and Topic	Item #	Checklist item	Location where item is reported
Abstract	2	See the PRISMA 2020 for Abstracts checklist.	3
**INTRODUCTION**			
Rationale	3	Describe the rationale for the review in the context of existing knowledge.	5
Objectives	4	Provide an explicit statement of the objective(s) or question(s) the review addresses.	5
**METHODS**			
Eligibility criteria	5	Specify the inclusion and exclusion criteria for the review and how studies were grouped for the syntheses.	7
Information sources	6	Specify all databases, registers, websites, organisations, reference lists and other sources searched or consulted to identify studies. Specify the date when each source was last searched or consulted.	7
Search strategy	7	Present the full search strategies for all databases, registers and websites, including any filters and limits used.	6-7
Selection process	8	Specify the methods used to decide whether a study met the inclusion criteria of the review, including how many reviewers screened each record and each report retrieved, whether they worked independently, and if applicable, details of automation tools used in the process.	6-8
Data collection process	9	Specify the methods used to collect data from reports, including how many reviewers collected data from each report, whether they worked independently, any processes for obtaining or confirming data from study investigators, and if applicable, details of automation tools used in the process.	6-8
Data items	10a	List and define all outcomes for which data were sought. Specify whether all results that were compatible with each outcome domain in each study were sought (e.g. for all measures, time points, analyses), and if not, the methods used to decide which results to collect.	6-7
	10b	List and define all other variables for which data were sought (e.g. participant and intervention characteristics, funding sources). Describe any assumptions made about any missing or unclear information.	6-7
Study risk of bias assessment	11	Specify the methods used to assess risk of bias in the included studies, including details of the tool(s) used, how many reviewers assessed each study and whether they worked independently, and if applicable, details of automation tools used in the process.	8
Effect measures	12	Specify for each outcome the effect measure(s) (e.g. risk ratio, mean difference) used in the synthesis or presentation of results.	8
Synthesis methods	13a	Describe the processes used to decide which studies were eligible for each synthesis (e.g. tabulating the study intervention characteristics and comparing against the planned groups for each synthesis (item #5)).	6-8
	13b	Describe any methods required to prepare the data for presentation or synthesis, such as handling of missing summary statistics, or data conversions.	6-8
	13c	Describe any methods used to tabulate or visually display results of individual studies and syntheses.	6-8
	13d	Describe any methods used to synthesize results and provide a rationale for the choice(s). If meta-analysis was performed, describe the model(s), method(s) to identify the presence and extent of statistical heterogeneity, and software package(s) used.	6-8
	13e	Describe any methods used to explore possible causes of heterogeneity among study results (e.g. subgroup analysis, meta-regression).	6-8
	13f	Describe any sensitivity analyses conducted to assess robustness of the synthesized results.	6-8
Reporting bias	14	Describe any methods used to assess risk of bias due to missing results in a synthesis (arising from reporting biases).	6-8

Section and Topic	Item #	Checklist item	Location where item is reported
assessment			
Certainty assessment	15	Describe any methods used to assess certainty (or confidence) in the body of evidence for an outcome.	6-8
**RESULTS**			
Study selection	16a	Describe the results of the search and selection process, from the number of records identified in the search to the number of studies included in the review, ideally using a flow diagram.	10-13
	16b	Cite studies that might appear to meet the inclusion criteria, but which were excluded, and explain why they were excluded.	10-13
Study characteristics	17	Cite each included study and present its characteristics.	10-13
Risk of bias in studies	18	Present assessments of risk of bias for each included study.	Suppl.mat.
Results of individual studies	19	For all outcomes, present, for each study: (a) summary statistics for each group (where appropriate) and (b) an effect estimate and its precision (e.g. confidence/credible interval), ideally using structured tables or plots.	Suppl.mat.
Results of syntheses	20a	For each synthesis, briefly summarise the characteristics and risk of bias among contributing studies.	Suppl.mat.
	20b	Present results of all statistical syntheses conducted. If meta-analysis was done, present for each the summary estimate and its precision (e.g. confidence/credible interval) and measures of statistical heterogeneity. If comparing groups, describe the direction of the effect.	10-13
	20c	Present results of all investigations of possible causes of heterogeneity among study results.	10-13
	20d	Present results of all sensitivity analyses conducted to assess the robustness of the synthesized results.	10-13
Reporting biases	21	Present assessments of risk of bias due to missing results (arising from reporting biases) for each synthesis assessed.	10-13
Certainty of evidence	22	Present assessments of certainty (or confidence) in the body of evidence for each outcome assessed.	Suppl.mat.
**DISCUSSION**			
Discussion	23a	Provide a general interpretation of the results in the context of other evidence.	13-15
	23b	Discuss any limitations of the evidence included in the review.	16-17
	23c	Discuss any limitations of the review processes used.	16-17
	23d	Discuss implications of the results for practice, policy, and future research.	16
**OTHER INFORMATION**			
Registration and protocol	24a	Provide registration information for the review, including register name and registration number, or state that the review was not registered.	6
	24b	Indicate where the review protocol can be accessed, or state that a protocol was not prepared.	6
	24c	Describe and explain any amendments to information provided at registration or in the protocol.	6

Section and Topic	Item #	Checklist item	Location where item is reported
Support	25	Describe sources of financial or non-financial support for the review, and the role of the funders or sponsors in the review.	17
Competing interests	26	Declare any competing interests of review authors.	-
Availability of data, code and other materials	27	Report which of the following are publicly available and where they can be found: template data collection forms; data extracted from included studies; data used for all analyses; analytic code; any other materials used in the review.	-

From: Page MJ, McKenzie JE, Bossuyt PM, Boutron I, Hoffmann TC, Mulrow CD, et al. The PRISMA 2020 statement: an updated guideline for reporting systematic reviews. BMJ 2021;372:n71. doi: 10.1136/bmj.n71

For more information, visit: http://www.prisma-statement.org/

Figure S10. Forest plot of all-cause mortality for the subgroup analysis of daily and non-daily reporting on the RTM

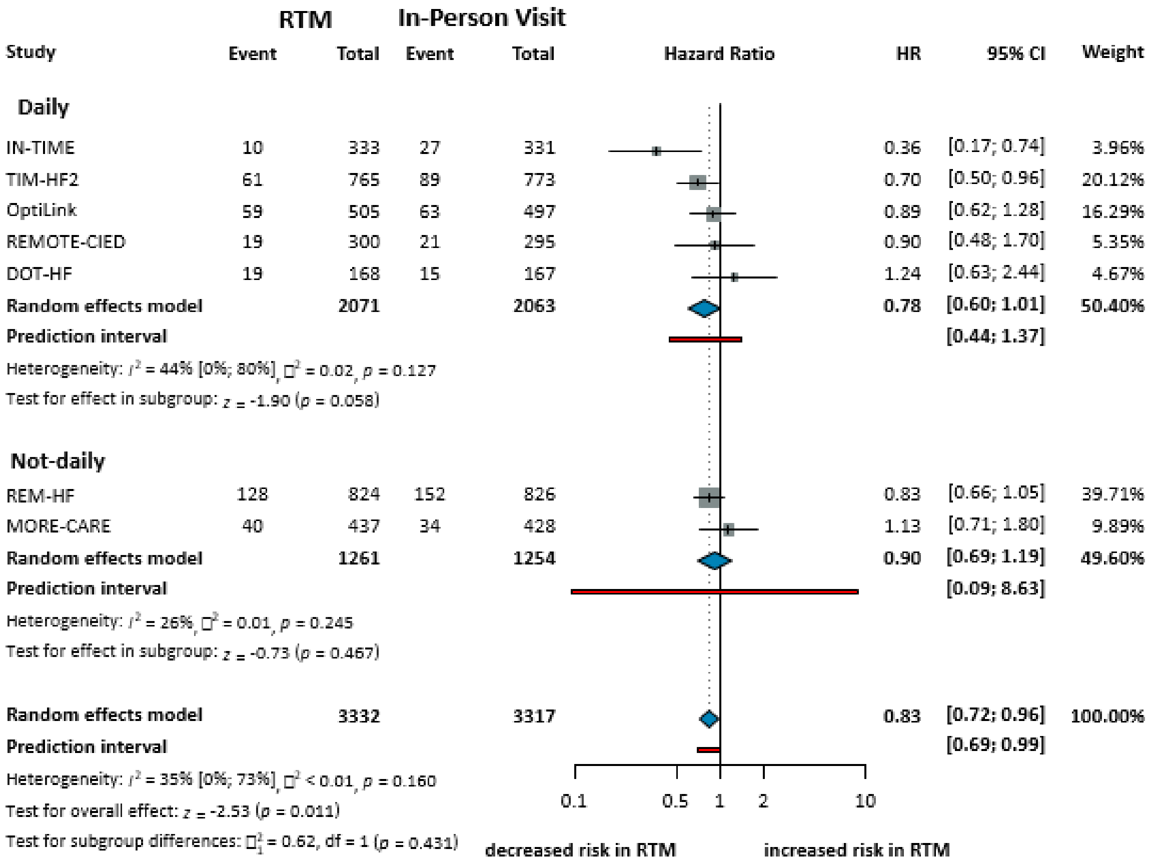

Figure S11. Forest plot of all-cause mortality for the subgroup analysis of regularity of in-office visits (3,6,12 months) on the RTM

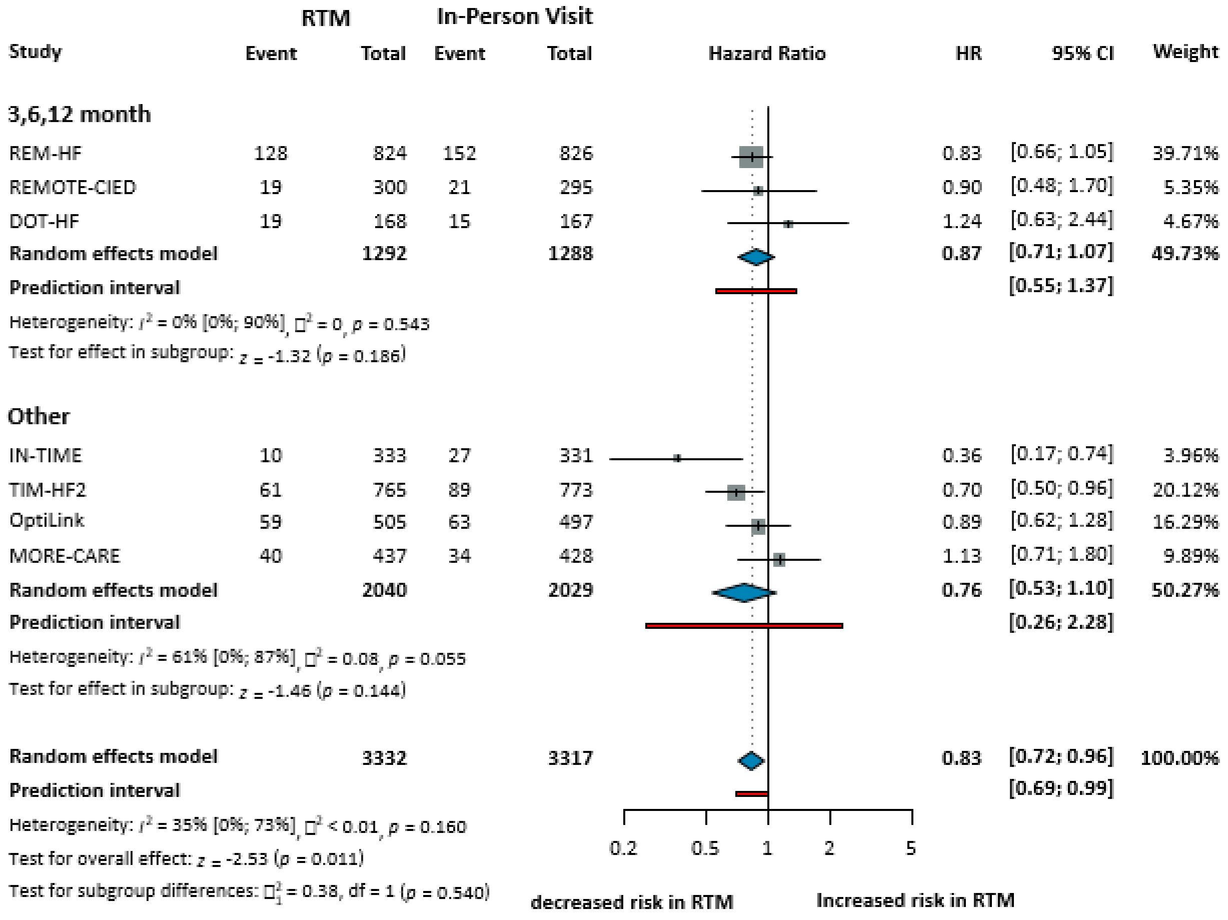

Figure S12. Forest plot of all-cause mortality for the sensitivity analysis based on Rob2 low and some concerns.

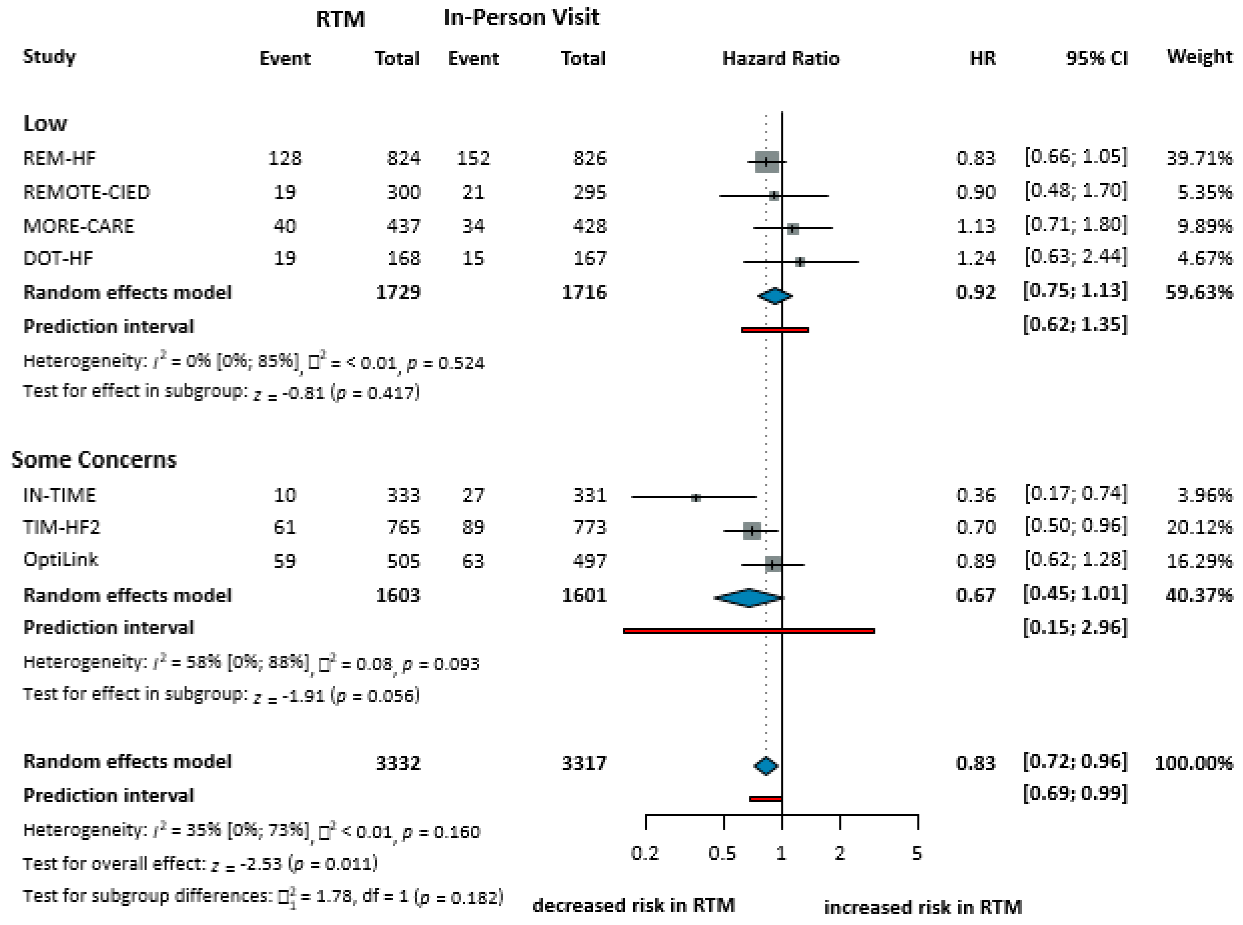

Table S1. Additional information about the trials

Author, year	Trial name	Number of patients		CIED				Follow-up (month)	Follow-up schedule		Design & Centers	Population & Criteria	Subgroup Effects	Adjudication	Endpoints
		RTM	IPV	RTM		IPV			RTM	IPV					
				CRT-D	ICD	CRT-D	ICD								
Varma 2010[19]	TRUST	908	431	0 (0%)	908 (68%)	0 (0%)	431 (32%)	15	Daily remote alerts for specific device or arrhythmia events (e.g., VT/VF, impedance issues, battery, mode switch, etc.). Clinic visits at 3 and 15 months. Interim checks done online every 3 months. Additional in-person visits allowed if remote checks deemed insufficient.	Home Monitoring disabled. In-clinic device interrogations every 3 months. Unscheduled visits allowed based on symptoms or physician request.	New ICD implants; adults	New ICD implants; adults	None	Yes	number of total in-hospital device evaluations
Al-khatib 2010[20]	pilot study	76	75	13 (17%)	63 (83%)	15 (20%)	60 (80%)	12	Remote monitoring every 3 months; clinic visit at 12 months; phone contact at 6 months; ad hoc visits as needed.	visit every 3 months and any time based on physician's choice	Single centre, prospective, pilot RCT	ICD and CRT-D adult patients	None	No	composite of CV hospitalizations, ER visits, and unscheduled device clinic visits at 1 year, medication use, QoL, cost, and patient satisfaction.
Veldhuisen 2011[21]	DOT-HF	168	167	141	27	133	34	15	Received full device-based	No fluid alerts provided;	Multicentre,pros	Adults with ICD/CRT-D and	None	Yes	Primary endpoint: all-cause mortality + HF

									diagnostics, including audible OptiVol fluid alerts Every alert required patient-clinician contact; care was guided by a standardized intervention algorithm. Clinical data were collected at 3, 6 months, and every 6 months thereafter, plus at unscheduled events.	patients knew alerts were off. Device integrity alerts remained active. Same follow-up schedule, but without access to OptiVol data or fluid notifications.	pective RCT	NYHA II–IV, HF hospitalization in past year. Excluded: recent cardiac surgery or MI, transplant listed, severe comorbidities, or limited life expectancy.			hospitalizations. Secondary: components of primary, outpatient visits, and OptiVol alerts linked to clinical events.
Crossley 2011[22]	CONNECT	1014	983	ICD and CRT-D patients 1:1 manner				15	In-office visits at 1, and 15 months. 3.6.9.12 months remote device transmission	In-office visits at 1, 3, 6, 9, 12, and 15 months post-implant.	Multicentre,prospective RCT	New ICD/CRT-D patients (1:1); excluded: permanent AF, warfarin use, life expectancy <15 months.	None	No	Time from clinical event to decision for arrhythmias, CV progression, or device issues.
Guedon-Moreau 2012[23]	ECOST	221	212	0 (0%)	221 (51%)	0 (0%)	212 (49%)	27	Follow-up at 1–3, 15, and 27 months post-implant; additional visits as needed (by HM alerts or on request).	Follow-up at 1–3, 9, 15, 21, and 27 months post-implant; additional visits if requested.	Multicentre,prospective RCT	Post-implant ICD recipients, mainly NYHA I-II, Exclusion: patients in NYHA IV class at the time of ICD implantation	Yes	Yes	≥1 major adverse event (MAE) — death (all-cause/CV), or procedure/device-related event.
Hindricks, 2014[10]	IN-TIME	333	331	190 (57%)	143 (43%)	200 (60%)	131 (40%)	12	Telemonitoring responses and office visits were investigator-driven, guided by	Follow-up followed European guidelines; only the 12-month visit was mandatory.	Multicentre,prospective RCT	CRT-D or dual-chamber ICD patients (NYHA II–III); excluded:	Yes	Yes	worsening of composite clinical score at 12 months, all-cause death and HF hospitalization (ITT population).

									standardized phone interviews.			severe valve disease, uncontrolled hypertension, AF, cardiomyopathies, pericarditis, or myocarditis.			
Lüthje 2015[24]	pilot study	87	89	44 (50.6%)	43 (49.4%)	44 (49.4%)	45 (50.6%)	15	In case of an automatic OptiVol alert trans-mission, a pre-specified clinical decision routine was activated	standard in-office visits were performed every 3 months	Single centre, prospective RCT	Adult patients with newly implanted or replaced ICD or CRT-D Exclusion criteria were permanent atrial fibrillation, a life expectancy ≤15 months, pregnancy.	None	No	OptiVol alerts, urgent visits, hospitalizations, Ventricular tachyarrhythmias and mortality
Boriani 2016[25]	MORE-CARE	437	428	437 (100%)	0 (0%)	428 (100%)	0 (0%)	24	alternated remote checks with in-office visits every 4 months	in-office visits every 4 months.	Multicentre, prospective RCT	Adult patients with newly implanted CRT-D Excusion: permanent AT/AF, breastfeeding/pregnancy, life expectancy less than 1 year	None	Yes	Composite of mortality, CV/device-related hospitalizations, healthcare use and costs (system/patient), and RM safety in CRT-D care.
Domenichini 2016[26]	LIMIT-CHF	41	39	25 (61%)	16 (39%)	28 (72%)	11 (28%)	12	remote monitoring+ visit every 3–6 months	Baseline study visit followed by routine HF clinic visits with clinical, weight, and lab assessments.	Single centre, prospective RCT	Adults with ICD/CRT-D, prior HF hospitalization, LVEF <50%, NYHA III, on optimal therapy. Excluded:	None	No	HF readmissions, total hospital/ED visits, unscheduled HF visits, NYHA class, MLWHF score, 6MWT distance, BNP levels.

												NYHA IV, lung congestion, device alerts, or advanced renal failure.			
Böhm 2016[27]	OptiLink	505	497	323 (64%)	182 (36%)	304 (61.2%)	193 (38.8%)	23	Auto fluid alerts with predefined intervention protocol.	follow-up visits every 6 months after ran-domization up to 18 months.	Multicentre, prospective RCT	ICD/CRT-D patients (NYHA II–III) with recent HF event, diuretic use, or elevated BNP. Excluded: dialysis, severe COPD, planned transplant.	Yes	Yes	Composite of death and CV/hospitalizations (all-cause, CV, HF) during follow-up.
Morgan 2017[28]	REM-HF	824	826	442 (53.6%)	275 (33.4%)	438 (53%)	276 (33.4%)	34	Weekly remote data review with scheduled contacts at 3, 6, 12, and 24 months.	Standard follow-up per site protocol with patient contacts at 3, 6, 12, 24 months, and study end.	Multicentre, prospective RCT	Adults with ICD/CRT-D/CRT-P; excluded if recent procedure/ surgery, tech barrier, pregnancy, listed for transplant, limited life expectancy, CIED issues, or non-English.	None	Yes	Time to first event: all-cause death or unplanned CV hospitalization. Other outcomes: all-cause, CV, and non-CV death; CV/non-CV hospitalization (planned/unplanned).
Hansen 2018[29]	INCONTACT	102	108	32 (31.4%)	70 (68.6%)	36 (33.3%)	72 (66.7%)	13	Device checks at 4 and 7 months could be remote. Scheduled in-clinic visits at 1 and 13 months included Packer score, QoL (MLHFQ), and medication review.	Same in-person visits at 1 and 13 months with assessments (Packer score, MLHFQ, meds). ICD/CRT-D checks done in clinic at 4 and 7 months. Adverse events and device data discussed at	Multicentre, prospective RCT	Patients with ICD and CRT-D,age 18–79, EF ≤35%, NYHA I–III, home setup for Merlin@home™. **Exclusion:** Mobitz II/III AV block, severe renal failure, life	None	No	Packer score worsening at 13 vs 1 month (HF death/hospitalization, NYHA, self-assessed health), mortality, HF events, arrhythmias, unscheduled visits, ICD therapies, and QoL changes (12 months)

									Optional 10-month follow-up; unscheduled checks possible.	all visits or calls.		expectancy <12 months, pregnancy, active participation in another trial, recent MI or coronary intervention (<3 months).			
Tajstra 2020[30]	RESULT	299	301	127 (42.5%)	172 (57.5%)	122 (40.5%)	179 (59.5%)	12	Remote data reviewed daily by staff. Alerts triggered phone check-ins and therapy adjustments. One clinic visit at 12 months. Audible alerts on.	In-person follow-ups at 3, 6, 9, and 12 months. Audible alerts also active.	Single centre, prospective RCT	NYHA II–IV HF patients, LVEF ≤35%,, new ICD/CRT-D with wireless capability (within 4 weeks), **Exclusion:** Age <18, inability to use device, <1-year life expectancy (non-CV), active device complications (e.g., infection, lead issue).	None	No	All-cause/CV death or hospitalization, HF events, MI, stroke, (un)scheduled outpatient visits, time to first visit, QoL at baseline and 12 months.
Pluta 2020[31]	TELEREH-HF	208	62	76 (36.7%)	131 (62.8%)	30 (48.4%)	31 (50%)	2.25	Patients underwent a 9-week hybrid telerehabilitation (1 week inpatient, 8 weeks home-based, 5×/week), including aerobic, respiratory, and resistance	Patients received standard care based on clinical routine with no structured rehabilitation or remote monitoring.	Multicentre, prospective RCT	Stable HF patients with ICD/CRT-D/CRT-P (NYHA I–III, LVEF ≤40%) recently hospitalized for HF within 6 months.	None	No	all-cause mortality, hospitalisations (all-cause, cardiovascular and due to worsening HF), number of alerts

									training. Sessions were guided by a remote ECG device and monitored by a clinical team. Daily adjustments were based on ECGs and symptom checks. Devices transmitted daily data and alerts to center staff; an experienced physician verified arrhythmias using device algorithms.						
Chiu 2022[32]	REMOTE-CIED	300	295	114 (38%)	186 (62%)	116 (39%)	179 (61%)	24	Remote ICD check-ups every6 months; in-person clinic visits at 12 and 24 months. Data collected remotely matched standard in-clinic interrogations.	Clinic visits every 3–6 months per site routine. ICD and alert programming was determined by each center.	Multicentre, prospective RCT	**Inclusion:** Patients aged 18–85 with symptomatic HF (NYHA II–III, LVEF ≤35%) and newly implanted Boston Scientific ICD compatible with LATITUDE ™ system. **Exclusion:** Awaiting transplant, major psychiatric /cognitive issues, or language barriers.	Yes	No	composite of all-cause mortality and cardiac hospitalization, mortality and cardiac hospitalization as independent endpoints and ICD therapy.
Koehler 2018[33]	TIM-HF2	765	773	Only the number of ICD and CRT patients was reported: 240 (15%) with CRT (including CRT-D and CRT-P) and 456 (29%) with ICD				12	Daily home transmission of vital signs (ECG, BP, HR,	Standard care by GP and cardiologist following	Multicentre, prospective RCT	**Inclusion**: Patients hospitalized for worsening	Yes	Yes	% of days lost due to unplanned CV hospitalization or all-cause death

						SpO_2, weight) to a telemedical center with 24/7 physician support. Risk stratification, monthly nurse calls, and medication adjustments as needed. Follow-ups at 0, 3, 6, 9, and 12 months.	HF guidelines. Same follow-up schedule as RPM group, without remote monitoring or centralized support.		HF within 12 months, NYHA II–III, LVEF ≤45% (or on diuretics if >45%). **Exclusion**: Major depression (PHQ-9 >9), haemodialysis, recent hospitalization (last 7 days), recent/scheduled LVAD, revascularization, TAVI, mitral clip, or CRT within ±28 days of randomization.			during follow-up, All-cause and CV mortality, % of days lost due to unplanned CV or HF hospitalizations, changes in MLHFQ score, NT-proBNP, and MR-proADM from baseline to study end.
